# Engineering pattern formation and morphogenesis

**DOI:** 10.1042/BST20200013

**Published:** 2020-06-08

**Authors:** Jamie A. Davies, Fokion Glykofrydis

**Affiliations:** Deanery of Biomedical Sciences and Centre for Mammalian Synthetic Biology, University of Edinburgh, U.K.

**Keywords:** morphogenesis, pattern formation, synthetic morphogenesis, synthetic morphology, tissue engineering

## Abstract

The development of natural tissues, organs and bodies depends on mechanisms of patterning and of morphogenesis, typically (but not invariably) in that order, and often several times at different final scales. Using synthetic biology to engineer patterning and morphogenesis will both enhance our basic understanding of how development works, and provide important technologies for advanced tissue engineering. Focusing on mammalian systems built to date, this review describes patterning systems, both contact-mediated and reaction-diffusion, and morphogenetic effectors. It also describes early attempts to connect the two to create self-organizing physical form. The review goes on to consider how these self-organized systems might be modified to increase the complexity and scale of the order they produce, and outlines some possible directions for future research and development.

## Introduction

This mini-review addresses early steps in engineering biological patterns and shapes, and sets out some ideas for future work. Given the limited space available, the article will focus on the bio-engineering of systems that make self-organizing patterns and shapes rather than, for example, on the direct bio-printing of biological structures (see [1] for a review of these). It will also focus strictly on mammalian systems, though the authors acknowledge that excellent work on these themes has also been done with bacteria and plants [2–6].

## Definition of key terms

The terms ‘pattern formation’ and ‘morphogenesis’ belong to the fields of embryology and developmental biology. They refer to two ways in which systems increase their complexity in the spatial domain. The two events operate on what developmental biologists call a ‘field’ — a volume of a cell, or more commonly of a population of cells, in which elements are connected by some kind of communication link which might be chemical, mechanical or even electrical. In pattern formation, an initially uniform field of cells acquires a non-random inhomogeneity, predictable either in detail (e.g. the pattern of bones in the hand) or predictable in statistical character even though not in detail (e.g. the pattern of a fingerprint, which is unique to individuals even in identical twins [7]). In the related phenomenon of ‘pattern elaboration’ a simple pattern in a field becomes more complicated as when, for example, an alternation of two cell states becomes a repeating series of three cell states. The cell states are usually states of gene expression so the result of a pure patterning event, with no morphogenetic mechanisms yet activated, will not usually be visible unless cells are stained to reveal the products of specific genes.

Patterning is often followed by morphogenesis, the creation of anatomical form. The differing cell states in the pattern activate cell behaviours, such as proliferation, adhesion, or folding, that drives the development of physical shape. The formation of ribs and the muscles between them, from an initially uniform sheet of pre-somitic mesoderm, is a clear example (albeit one that proceeds by many stages). Sometimes, patterning is made visible through non-morphogenetic processes; hair colour, as in leopard spots or tiger stripes, is a well-known case. Also, sometimes morphogenesis takes place without prior patterning, as in the purely mechanical folding of the chick intestine [8]. Generally, though, animal development proceeds by the alternation of patterning, morphogenesis and growth. After growth has expanded a patterned tissue, its subcomponents may pattern again to create finer details. So we begin with coarse patterns (head end, tail end) then finer ones (skull, jaws, neck, thorax…) then finer still (teeth, individual cusps on teeth…) and so on. As a general rule, albeit with some exceptions, most patterning events take place over spatial scales of the order of 100 µm, presumably because that is a spatial scale over which protein gradients are steep enough to be accurately readable by cells [9].

## Why engineer patterning and morphogenesis?

There are two main incentives to engineer pattern formation and morphogenesis, one belonging to basic science and the other to biotechnology. Traditionally, the study of developmental biology has been predominantly analytic; researchers observe embryos growing naturally, form hypotheses about how specific aspects of development work, perturb the system (for example by knocking out a gene) and analyse the resulting change in the developmental trajectory. This approach results in vast detailed specific knowledge, but drawing and testing principles from the data is not straightforward. Testing a system by knocking components out does not confirm that the system is completely understood. While knocking out components of a system suffices to test necessity, understanding the axiomatic principles for sufficiency requires the bottom-up approach offered by synthetic biology. By analogy, one could form a theory of helicopter flight based on the engine, the transmission and the main rotor, ‘knock out’ any of the three and observe the crash, and conclude the theory is correct without ever suspecting the vital role of the tail rotor. But if one tried to build a helicopter from first principles, the rapidly spinning resultant mess would make it immediately obvious that something critical was missing; adding the tail rotor to make a better version would lead to an understanding of its function, as would replacing it with an alternatively-engineered solution (e.g. a small horizontal jet engine). It is in this spirit that engineering patterning and morphogenesis is of service to our basic understanding of development [10]; if our theories are correct, we should be able to build a working system from first principles. If we fail, we need to go back to the embryo with new questions informed by our failure.

The other reason for wanting to engineer patterning and morphogenesis is to construct tissues, either as engineered close copies of natural tissues or brand new ‘designer tissues’. Such tissues could be intended, for example, to function in extracorporeal life-support machines, or to be a custom component to repair an atypically formed body, or even to be a substitute for animal tissues, for example for ‘vegetarian meat’ [11]. In this context, engineering pattern formation and morphogenesis becomes an asset for tissue engineering in general.

## Engineered pattern formation

Patterning systems typically involve architectures of cell–cell communication that include short-range stimulation and long-range inhibition. The first-proposed mechanisms for this were the reaction-diffusion systems proposed by Turing [12] and, later, by Meinhardt [13]; both versions use diffusion-mediated chemical signalling. A synthetic version has been built and will be described later in this section, but the earliest synthetic patterning systems used contact-driven, rather than diffusion-mediated, processes to mediate short-range stimulation and long-range inhibition, so we will describe these synthetic systems first.

**Figure 1.**
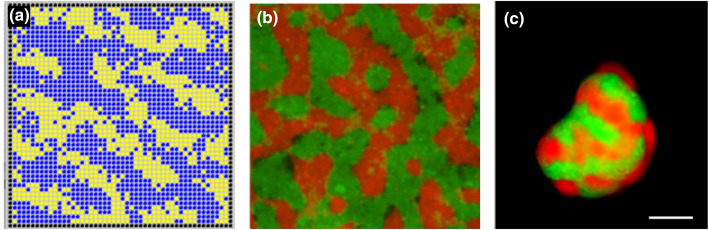
Patterning by phase separation. (**a**) Computer model of yellow and blue cells, each of which adheres strongly to cells of its own but not the other colour. The simulation begins with randomly placed cells, and allows cells to swap position, one at a time, if the swap reduces free energy of the system; the result generates a pattern of patches. (**b**,**c**) *in vivo* recreation of phase separation using Cdh1 and Cdh3 homophilic adhesion systems in Hek293 cells in (**b**) 2-dimensions and (**c**) 3-dimensions. These images come from image sets used in the writing of our experimental report on the study [18]; scale bar 200 µm.

The first mammalian synthetic biological patterning system depended on constrained cell sorting driven by differential adhesion. It has been known since the 1960s [14–17] that two cell types, A and B, with different adhesivities, will sort so that the most mutually adhesive cells will cluster in the centre of a clump, surrounded by the less adhesive type. The behaviour was explained on thermodynamic grounds of minimizing free energy (of unoccupied potential adhesion sites), as an example of phase separation analogous to the phase separation of a mixture of oil and water into separate layers. While it is now known that active cell biological responses to adhesion are also involved [18,19], the simple thermodynamic model remains adequately predictive. Computer modelling of this phenomenon in the context of a 2-dimensional cell sheet (by one of the current authors) suggested that phase separation of homotypically adhesive A cells and homotypically adhesive B cells would be incomplete and would result in a pattern of patches. Essentially, when A-type cells encounter one another they stick to make a patch of A-type cells, and the depletion of A-cells from the surrounding area leaves B-cells separating one A-cell patch from the next. While free energy would be minimized by all A-patches coalescing, cells cannot move to achieve this because movement of A-cells into areas of B-cells would be energetically disfavoured. The result is a patchwork, both in theory (Figure 1a) and also, when realized by engineering expression of different inducible cadherin-type homophilic adhesion molecules into HEK cells, making random mixes of the cells and then inducing cadherin expression, in practice (Figure 1b) [20]. The spontaneous generation of patterns from random mixes of cells shown in 2-dimensions in Figure 1b also occured in 3-dimensional clumps (Figure 1c).

**Figure 2.**
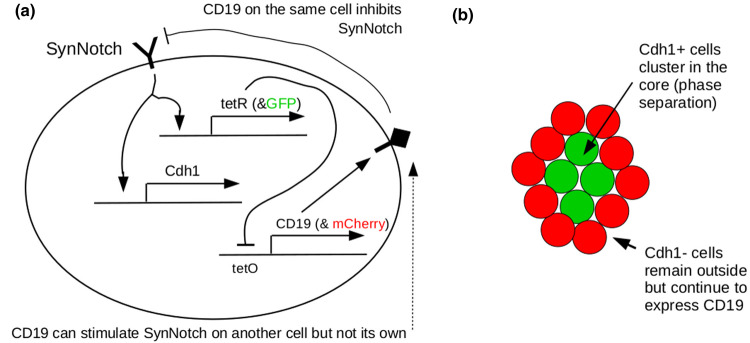
The Syn-Notch-mediated patterning system of Toda and colleagues [20]. (**a**) depicts the gene circuit in one cell (the outer oval represents the plasma membrane), and (**b**) depicts emergent multicellular behaviour. Cells adopt one of two fates according to the relative level of CD19-triggered SynNotch signal. This level depends weakly on noise and strongly on the state of their immediate neighbours. Having chosen a fate, the cells sort by Cdh1-mediated phase separation into two layers.

A different type of contact-mediated patterning was constructed using the SynNotch signalling system [21], a synthetic biological construct based on natural Notch-Delta signalling [22]. This pattern again used adhesion, but now the pattern emerged from a single cell type rather than a mixture. Cells were engineered to express the adhesion molecule Cdh1 and the transcriptional repressor TetR in response to SynNotch detecting its ligand, CD19, on a different cell (activation in trans and repression in cis is a feature of Notch signalling). The cells also expressed CD19, under the control of a normally active promoter inhibited by TetR. This effectively gave the cells two states; not receiving an external CD19 signal, thus expressing CD19 but not Cdh1, or receiving external CD19, thus not expressing CD19 but expressing Cdh1. Cells cultured densely enough to experience cell-cell contact spontaneously split into two populations, with the two available cell states. Because of their different expression of Cdh1, cells sorted into two layers (Figure 2).

**Figure 3.**
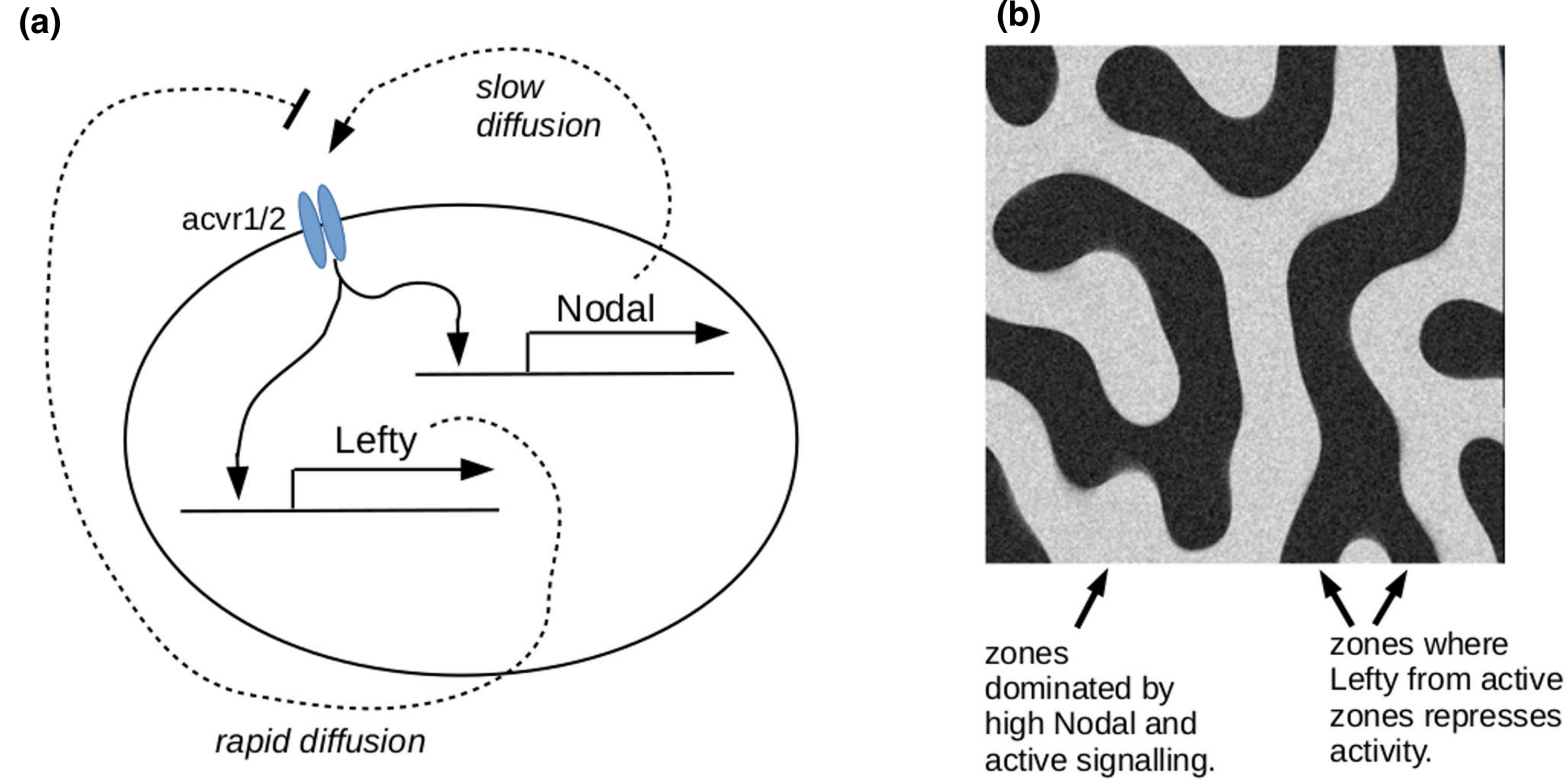
The reaction-diffusion patterning system of Sekine and colleagues [21]. (**a**) depicts the genetic circuit responsible for generating short-range activation and long-range repression. (**b**) depicts the type of pattern formed (from a computer model written by one of the current authors).

One system has been constructed using the architecture of the Turing system (or at least something very close) [23]. Cells were engineered to express both the short-range signalling molecule, Nodal, and the long-range Nodal inhibitor, Lefty, in response to signalling through Nodal's Acvr1/2 receptor complex. Cells with active Acvr1/2 signalling produced Nodal, which accumulated in the area and recruited some neighbouring cells into activation, but these activated cells also became a source of Lefty. This diffused away to create more distant zones dominated by Lefty's inhibitory influence. Only beyond those zones could new centres of activation develop (Figure 3). The Nodal/ Lefty system is used for natural patterning in embryogenesis: setting up synthetic systems from scratch, with components that have not evolved for the task, has proved more challenging and has focused attention on key elements of design [24,25].

A form of pattern formation seen in embryos, but not yet in synthetic form in animal cells, is the clock and wavefront mechanism which can be used to subdivide a large field into repeating segments. Here a ‘wave’ of activation travels across a field of cells, which are also subject to an oscillating clock (internally generated or externally imposed), the clock completing many cycles in the time it takes the wave to pass across the field. Every time the clock ‘ticks’, cells that are in the active state are induced to change in some way (for example by transcribing a new, self-stabilizing, combination of genes). The result is be a series of stripes across the field, each recording the position of the wave of activation at different ticks of the clock.

## Pattern elaboration

**Figure 4.**
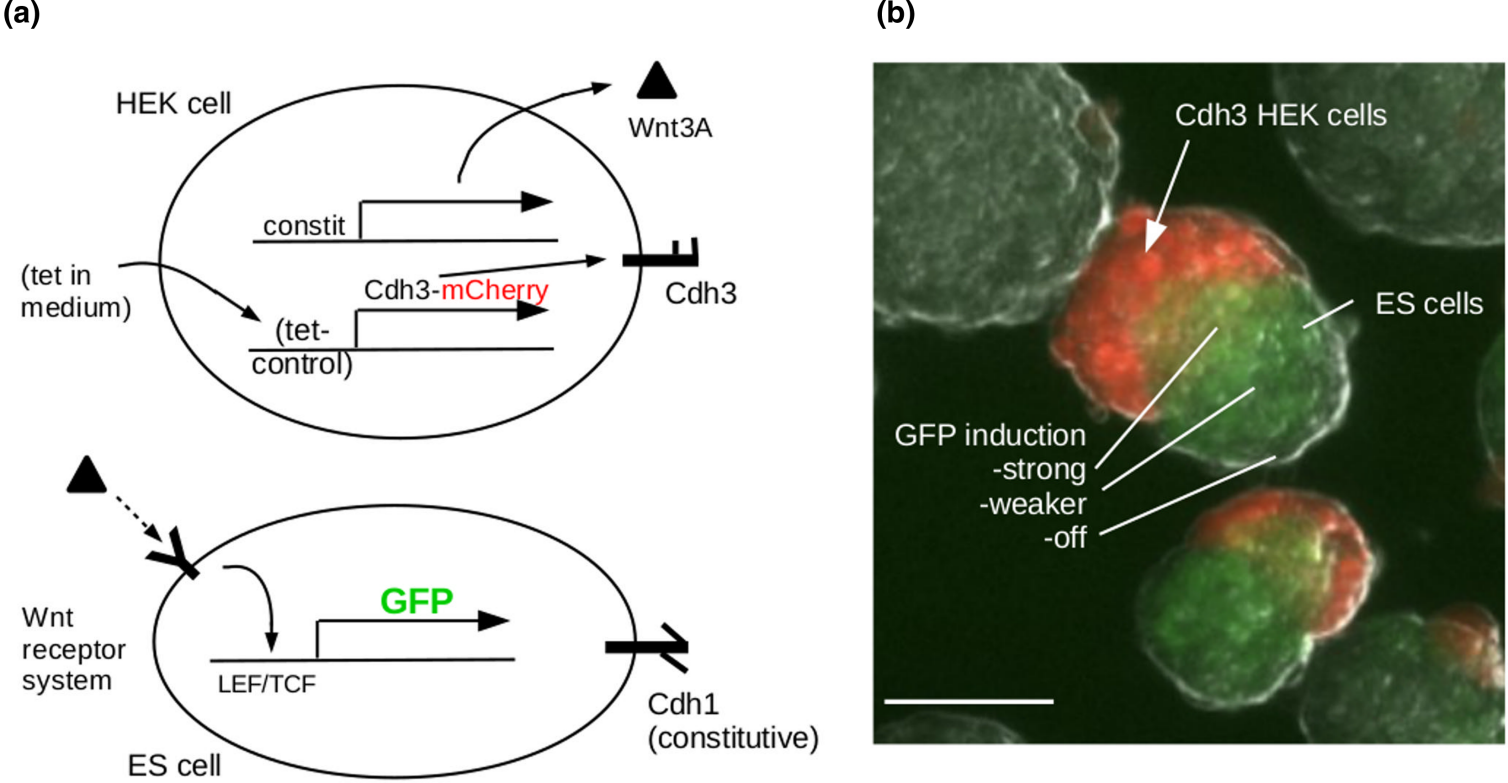
Pattern elaboration. (**a**) shows the genetic constructs and (**b**) the behaviour, in which red and green cells sort by phase separation and Wnt signalling from red cells induces a gradient of GFP expression in the ES cell (Chd1) phase. Authors’ unpublished data. Scale bar 100 µm.

Pattern elaboration is the process by which a simple pattern acquires further complexity. This can happen in a variety of ways but one of the most straightforward is to exploit the fact that the primary patterning event generates juxtapositions of zones with different states, and a cell finding itself at such a boundary can then be triggered to enter an additional state not entered by cells far from boundaries. We have constructed a simple demonstration of this idea, by mixing Cdh3-expressing cells that also express a Wnt signalling molecule, with Cdh1-expressing cells that become GFP+ in response to Wnt signals. The Cdh1 and Cdh3-expressing cells sort by phase separation to generate two zones, as described before, and the Cdh1-expressing cells nearest to the Wnt3A-expressing cells become GFP+, generating a third state (Figure 4). Indeed, the GFP expression is not just a single state but forms a gradient that could be used to organize further sub-patterns.

In natural embryos, pattern elaboration often follows earlier patterning only after a period of growth that expands a field. This has not yet, to our knowledge, been explored in synthetic systems, partly for the technical reason that overall growth is not easily compatible with the commonly used technique of culture on tissue culture plastic. Suspension culture, such as was used for Figure 4, will allow growth that is unconstrained by cell attachment to anything external to the culture. It may therefore be possible to pattern a suspension-cultured system, allow it to grow larger by the normal process of cell proliferation, and then invoke a new round of patterning, by a new mechanism, in each of the now larger domains that were formed in the first round.

## Engineered morphogenesis

Fortunately, many aspects of natural morphogenesis rest on a relatively small number of cell behaviours, expressed in different combinations, orders and extents in different developing systems. These behaviours include proliferation, death, fusion, motility, adhesion and clumping (condensation), mesenchyme-to-epithelial transition, epithelial folding, epithelial-to-mesenchyme transition, and neighbour exchange [19,26]. Following the publication of a 2008 article on the potential for synthetic morphogenesis [27], Cachat and colleagues constructed a library of DNA modules that drive typical mammalian cell lines to exhibit many of these behaviours (separately, and for cell lines, proliferation control was in the direction of stopping the proliferation that is their default state) [28].

**Figure 5.**
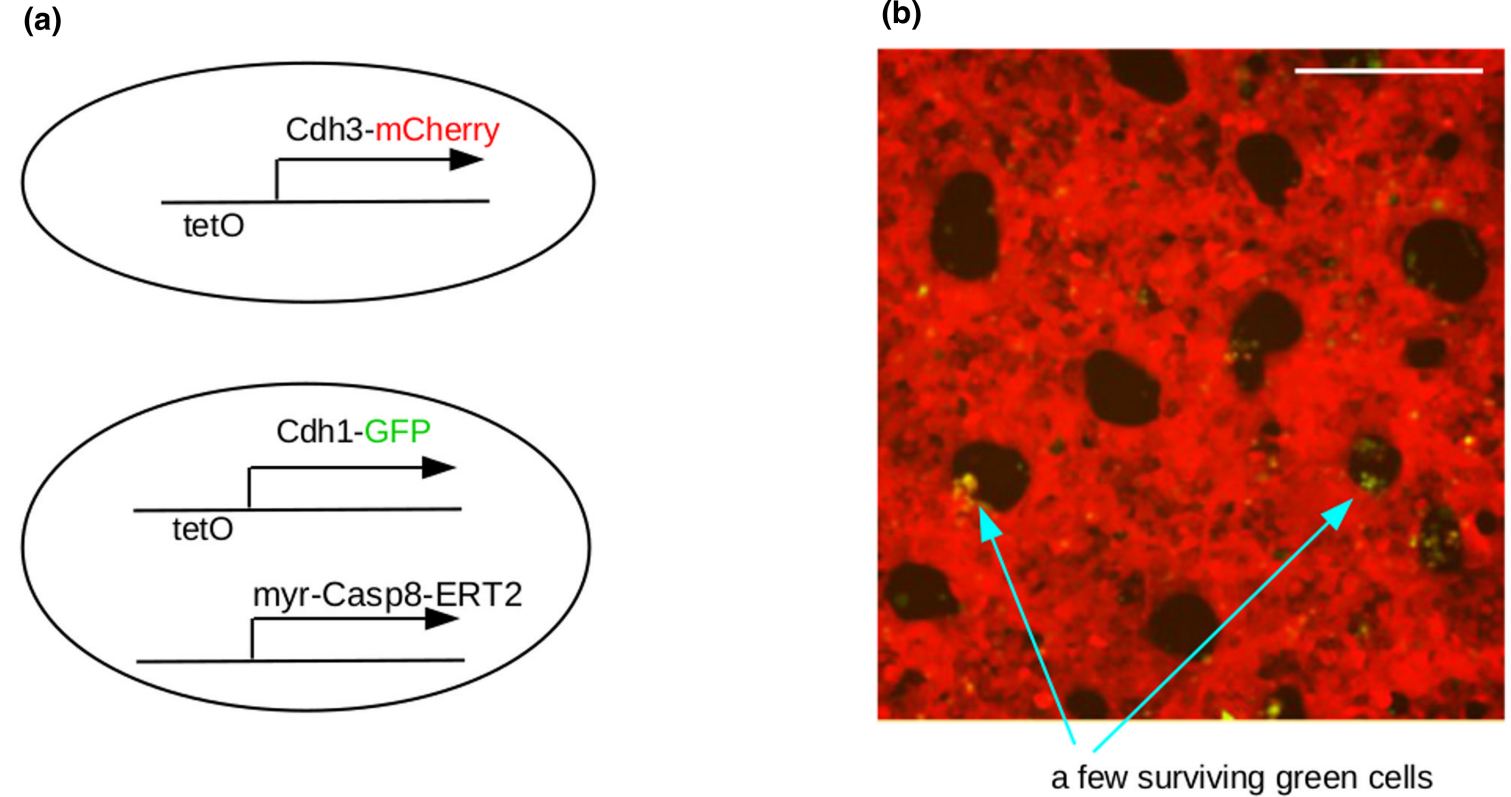
Patterning followed by morphogenesis. (**a**) shows the genetic constructs in the two cell types. The cells generated a phase separation pattern on induction of the adhesion systems with tetracycline, to inhibit the TetR repressor protein that is constitutively expressed in these cells. Then, when tamoxifen is added to the medium, the Caspase8-ERT2 induces apoptosis in the Cdh1, green cells, to leave holes in a sieve-like ‘tissue’. Data are from our image sets for [25]; scale bar 200 µm.

The function of the modules was initially demonstrated in the context of very simple verification assays. More recently, however, there has been the beginning of a connection between patterning systems and the morphogenetic modules. In one example, patterning by phase separation generates patches of one phase amongst cells of the other phase, then a cell death programme is induced in the patches to leave a sieve-like sheet of cells with holes (Figure 5) [29]. Work is now underway to couple patterning to more sophisticated morphogenetic responses than simple elimination.

A completely different approach to engineered morphogenesis is the ‘reverse engineering’ approach epitomised by Kit Parker's group in Harvard. In a striking experiment [30], they ‘reverse engineered’ a swimming jellyfish by assembling a sheet of rat cardiomyocytes on a polydimehtylsiloxane flower-shaped ‘bell’, with surface modifications of the bell controlling the orientation of the cardiomyocytes. Electrical stimulation of the culture bath caused the muscles to contract and to change the morphology of the bell, reversibly, so that it would return to its original shape on muscle relaxation. Thus a succession of pulses caused the bell to undergo repeated cycles of deformation and to ‘swim' in a manner similar to a jellyfish. This type of engineering, involving careful production of a patterned physical substrate and the use of highly specialized, differentiated cells is very different from the self-organizing approaches described earlier but it is still engineering of morphogenesis. Importantly, too, it indicates what might be done if self-patterning systems, such as those described in the rest of the current article, were to be coupled to sophisticated cell behaviours such as cell orientation and synchronized muscle contraction.

## Higher orders of patterning

**Figure 6.**
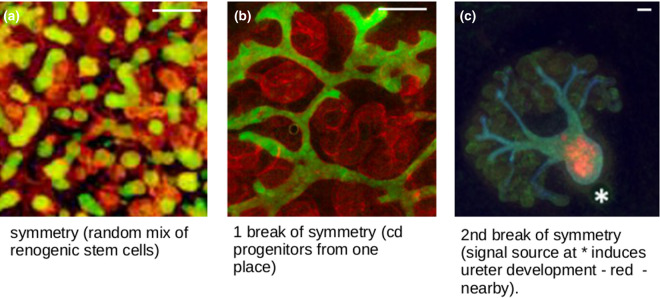
The importance of symmetry-breaking in generation of large-scale structures in organoids. (**a**) shows a renal organoid made from a random mix of renogenic stem cells; there are red developing nephrons and many small green developing collecting duct treelets scattered throughout. (**b**) shows the more realistic anatomy (single ramifying collecting duct tree) that results from introducing collecting duct progenitors at one location only. (**c**) shows how introducing a second break of symmetry, a local source of the signalling molecule BMP4, introduces large-scale polarized organization with a ureter (orange) at one end and kidney-type anatomy (blue collecting duct, green nephrons) at the other. Image c is from one of our images in [32], cc-4.0 licence. Scale bar 100 µm.

The types of patterning that have been described so far include small-scale features that are repetitive (statistically, even if not precisely) but no large-scale features. This is common in self-organizing systems such as organoids. In these, stem cells treated to differentiate towards particular fates of interest produce accurate representations of target tissues (e.g. renal) at the micro-scale but fail to produce the gross anatomical arrangement of the organ (reviewed in [31]). In a kidney organoid, for example, the even mix of cells means that many small urine collecting duct trees form, instead of the one single tree around which a normal kidney's tissues are arranged (Figure 6a) [32–34]. We have been addressing this problem in relation to the goal of turning simple renal organoids into useful kidneys, with a view to eventual transplantation. We find that the key to producing better large-scale anatomy lies in breaking the symmetry of the system. By restricting progenitors of the urine collecting duct system to be in just one location within the other cells, for example, we can drive the formation of organoids based much more realistically around one single tree Figure 6b [35]. Furthermore, by imposing an asymmetric external signalling environment, we can induce one end of that tree to differentiate into the type of tube found in the ureter (the urine exit of the natural kidney), while the kidney-type development takes place at the other end of the culture, increasing realism still further Figure 6c [36].

**Figure 7.**
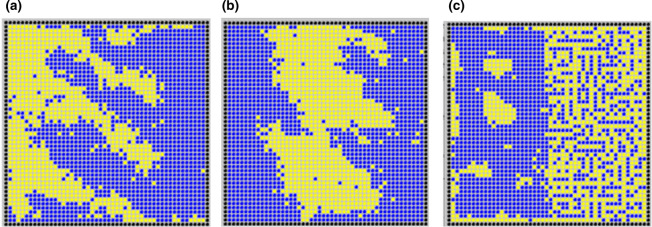
Simple simulations of the effect on patterning by phase separation, of making a parameter responsive to a large-scale gradient. In (**a**,**b**) the strength of homotypic adhesion is proportional to the concentration of a substance forming a linear gradient from (**a**) the left edges and (**b**) the centre of the culture. The scale is the same as in Figure 1a; the result is more efficient sorting to form larger patches, with ‘fingers’ emerging from it. In (**c**), homophilic adhesion is unaltered but heterophilic adhesion increases with distance from the left edge; the result is a change from patch production to very fine-scale mixing of different cell types; both parts are patterned, in the sense that they have a degree of order. To our knowledge, phase separation patterning systems with gradient-controlled parameters have not yet been built in real cells (simulation produced for this review).

The point of mentioning this organoid work here is that it illustrates clearly the way in which even very simple asymmetrical influences can cause self-organizing systems to generate anatomical order at large scales as well as at micro-scales. In principle, this idea might be extended to engineered self-patterning systems. Even the simple systems described above might have key parameters (production of an adhesion molecule, or production of one of the signalling molecules in a reaction-diffusion system) placed under the additional control of a long-range, short-lived diffusible molecule. Having a source of this molecule on one side of the field of cells would create a large-scale concentration gradient across it, and thus impose macroscopic organization on the dynamics of micro-scale patterning (Figure 7).

## Future directions

The synthetic biology field reviewed herein, albeit young, offers numerous possibilities for future research. One direction would be to create and test new systems of self-organization in cell lines *in vitro*, expanding the patterning toolbox. Through different approaches, some of which described in this review, simple ‘Version1.0' patterns might be modified and elaborated to increase pattern complexity, whether that relates to the amount of cell states present, to geometrical components and topology of the pattern, or to evolvability and robustness. The morphogenetic toolbox can be similarly expanded, and tying more interesting morphogenetic modules to different patterns might be used to explore questions that traditional developmental biology cannot answer. A very different direction would be to transfer engineered systems *in vivo*, for example to simple invertebrate embryos (C. elegans), to understand how they operate in a complex whole-organism background. All these approaches, and many more, will benefit from sophisticated computer modelling which might be used to predict network behaviour (to build complex circuits), or perform sensitivity analyses to identify parameters that require tight control (to create more robust systems). Progress in these fields is already being made.

Early benefits of this bottom-up approach will include improved understanding of axiomatic principles of embryonic development. With time, the generation and elaboration of synthetic patterning and morphogenetic systems will allow us to test more ideas. As knowledge, tools and technologies improve, the potential for applying patterning and morphogenetic systems to real-world problems, such as (synthetic) tissue engineering, will become clearer.

## Perspectives

- Engineering cells to undergo autonomous pattern formation and morphogenesis is important both for testing axiomatic understanding of developmental biology, and as a foundation for advanced tissue-engineering.
- Current work is laying the foundations for the field, by building or elaborating simple systems of pattern formation and morphogenesis, and performing proof-of-concept experiments that link the two.
- Advances in the field will require researchers to apply self-organization at different scales, add fine-tuned sophisticated levels of control, and engineer built-in robustness for pattern formation and final morphology.

## Open Access

Open access for this article was enabled by the participation of University of Edinburgh in an all-inclusive *Read & Publish* pilot with Portland Press and the Biochemical Society under a transformative agreement with JISC.
